# Domestic Correspondence

**Published:** 1882-08

**Authors:** 


### Domestic Correspondence.

ARTICLE XII.

LANSING, MICHIGAN, JULY 12, 1882.

*To the Honorable the Senate and House of Representatives, in United States Congress assembled:*

Your memorialist, the Michigan State Board of Health, respectfully represents:

That small-pox, diphtheria and scarlet fever have been and are being repeatedly introduced into this State by immigrants newly arrived from foreign infected places, and by travelers who have come in contact with such immigrants; that because of the rapidity of travel, and the vast amount of inter-State travel, it is impossible for State or local boards of health, without extraordinary interference with inter-State commerce, to successfully quarantine against or effectually control these diseases while the United States Government permits one or more of them to be introduced so frequently, as of late, by immigrant vessels which reach this country; that while in ordinary years the introduction of scarlet fever and diphtheria is believed to be of exceeding consequence in causing epidemics, in swelling the death-rates, sickness-rates, pauperism and general suffering, cholera and yellow fever are sometimes thus introduced, and at the present time small-pox is causing especially widespread disaster in this and other Western States; that, by reason of such introduction of disease, the lives, health, and happiness—those dearest and most important interests of our people—are constantly destroyed or placed in imminent danger from those foreign pestilences from which it is entirely possible for the United States Government to afford protection; that we believe that it is the highest duty of a government to protect the lives of its citizens from dangers which which threaten all, and from which no other than governmental protection is adequate; that it is with deepest apprehension that this State Board of Health learns that by reason of insufficient provisions, in the Sundry Civil Service Appropriation Bill, for the National Board of Health, the important work which that Board has lately commenced and is expected to do is likely to be crippled; that this Board believes that now, more than ever before, is a most inopportune time to lessen in any way the activity and usefulness of the National Board of Health, believing, as this Board does, that there is no other governmental department, bureau, or "service" so closely connected with the highest interests of all citizens and of humanity.

Therefore this State Board of Health earnestly prays Congress to grant sufficient appropriations to the National Board of Health, and to make such other provisions as will enable it to continue the immigrant-inspection service at all important ports of entry, and on important lines of travel, and to provide in every possible way for the protection of the whole country from cholera and yellow fever, and also from those contagious diseases hereinbefore mentioned, which are well-known as causing the most deaths and distress, throughout the greater portion of this country.

By direction and on behalf of the Michigan State Board of Health.

LE ROY PARKER, *President.*
HENRY B. BAKER, *Secretary.*

---

### ARTICLE XIII.

MONTICELLO, FLA., JULY 12, 1882.

### PREMATURE LABOR, INDUCED BY QUININE.

EDITORS CHICAGO MEDICAL JOURNAL AND EXAMINER:—The patient, æt. twenty-two, was five months gone in her second pregnancy; was in a debilitated and anæmic condition from repeated attacks of malarial fever.

Quinine had been given by her husband, in small doses, for some days, but failing to check the fever, he increased the dose to grains xiij, which was followed in ten hours by an abortion, with considerable hæmorrhage.

Yours very respectfully,

THEODORE TURNBULL, M.D.

---

### ARTICLE XIV.

CHICAGO, JULY 1, 1882.

MESSRS. EDITORS:—I have received a number of letters from physicians, asking for the strength of the yellow ointment, and I find that by some accident the formula was left out in the copy of the MS. To remedy this oversight, please state in a note in the next number of the JOURNAL AND EXAMINER, that the yellow ointment I use contains thirty grains of yellow oxide to ℥i vaseline.

Yours truly,

F. C. HOTZ.

---

### A CASE OF RARE DYSTOCIA BY TRANSVERSE OCCLUSION OF THE UTERUS.

The woman, thirty-nine years of age, had borne a child eighteen years ago. When pregnant again after this time she had very severe labor pains, but without any effect. The examination showed a membrane separating the cavity of the uterus from the cervix, which latter was voluminous, and as large as a silver dollar. The posterior cul-de-sac of the vagina was pressed downward, which indicated a sac-like dilatation of the posterior segment of the uterus. The membrane was cut with scissors, but the cavity of the uterus could not be entered. The woman died three hours afterward. The autopsy showed a placenta prævia, the child already putrefied. The cavity of the uterus was separated from the cervix by a membrane, in a transverse direction, and three centimeters long.—*Le Praticien.*

